# The bacterial burden of worn face masks—observational research and literature review

**DOI:** 10.3389/fpubh.2024.1460981

**Published:** 2024-12-03

**Authors:** Kai Kisielinski, Barbara Wojtasik, Aleksandra Zalewska, David M. Livermore, Agata Jurczak-Kurek

**Affiliations:** ^1^Clinical Medicine (Surgery), Emergency Medicine and Social Medicine, Private Practice, Düsseldorf, Germany; ^2^Department of Evolutionary Genetics and Biosystematics, Faculty of Biology, University of Gdansk, Gdansk, Poland; ^3^Norwich Medical School, University of East Anglia, Norwich, United Kingdom

**Keywords:** 16S rRNA gene amplicon sequencing, adverse effects, bacterial contamination, Rose Bengal staining, surgical mask, N95, personal protective equipment, risk

## Abstract

**Introduction:**

Facemasks were widely mandated during the recent SARS-CoV-2 pandemic. Especially the use by the general population is associated with a higher risk of improper handling of the mask and contamination and potential adverse microbiological consequences.

**Methods:**

We investigated and quantified bacterial accumulation in facemasks used by the general population, using 16S rRNA (Sanger Sequencing), culture and biochemical analysis along with Rose Bengal staining. Additionally, a systematic overview of the literature on face mask contamination was undertaken.

**Results:**

We found an average bacterial load of 4.24 × 10^4^ CFU recovered/mask, with a maximum load of 2.85 × 10^5^ CFU. This maximum is 310 times higher than the limit value for contamination of ventilation system outlet surfaces specified by the German standard VDI 6022. Biochemical and molecular identification predominantly found *Staphylococcus species* (80%), including *Staphylococcus aureus*, along with endospore-forming Bacillus spp. Literature reports also indicate contamination of masks by bacterial and fungal opportunists of the genera *Acinetobacter, Aspergillus, Alternaria, Bacillus, Cadosporium, Candida, Escherichia, Enterobacter, Enterococcus, Klebsiella* (including *K. pneumoniae*), *Micrococcus, Microsporum, Mucor, Pseudomonas, Staphylococcus and Streptococcus*. Bacterial counts increase linearly with wearing duration.

**Discussion:**

Prolonged use may affect the skin and respiratory microbiomes, promoting consequential eye, skin, oral and airway conditions. These aspects underscore the urgent need for further research and a risk-benefit analysis in respect of mask use, particularly given their unproven efficacy in disrupting the transmission of respiratory viruses and their adverse social consequences.

## Introduction

Facemasks covering the entrances to the airways were widely mandated during the recent SARS-CoV-2 pandemic, not only for healthcare workers but also for the general population (1). Professions with frequent human contact were obligated to wear them for long periods as were schoolchildren (1–6).

This raises reasonable concerns: first, because use by the general population is associated with a higher risk of improper handling of the mask (7–11); secondly because their efficacy against respiratory viral infections is unproven by high quality trials, which indicate little or no effect (12, 13) and thirdly, because masks are assumed only to have positive effects (14–16). In reality there is strong evidence that masks pose various risks, especially for pregnant women, children and adolescents, as well as older adults and the unwell (14, 16–19). They have several demonstrably adverse effects, affecting physiology (14, 16, 19–23), psychology (16, 24) and, most obviously, social interactions (25–35). Effects on childhood development are a particular concern. These adverse effects have been recently summarised as the so-called mask-induced exhaustion syndrome MIES (14, 16, 19). Interestingly, Spira (36) and Fögen (37) found significantly higher SARS-CoV-2 infection and mortality rates in the mask-wearing cohorts: explanations are uncertain, but viral trapping and recycling are plausible.

A further concern, encompassed within MIES, relates to the potential adverse microbiological consequences of wearing face masks. Owing to the creation of a warm, moist micro-environment (38–41), bacteria, fungi and even viruses may accumulate on both sides of the worn masks (42–46). So far, these aspects have not been evaluated in depth. The aim of our pilot study was to assess, visualise and categorise the general ability of masks to accumulate bacteria when used by the general population. This also with regard to a risk assessment, using worst-case consideration which is necessary in such a protective approach (47). Accordingly, we undertook a microbiological exploration with random samples of face masks as used by members of the general population, together with a systematic rapid literature review. This combined holistic approach with 16S rRNA (Sanger sequencing), culture and biochemical analysis along with Rose Bengal staining plus systematic literature analysis has not been performed before and is the first of its kind.

## Materials and methods

### Rose Bengal staining and visualisation of contamination

**Figure 1:**
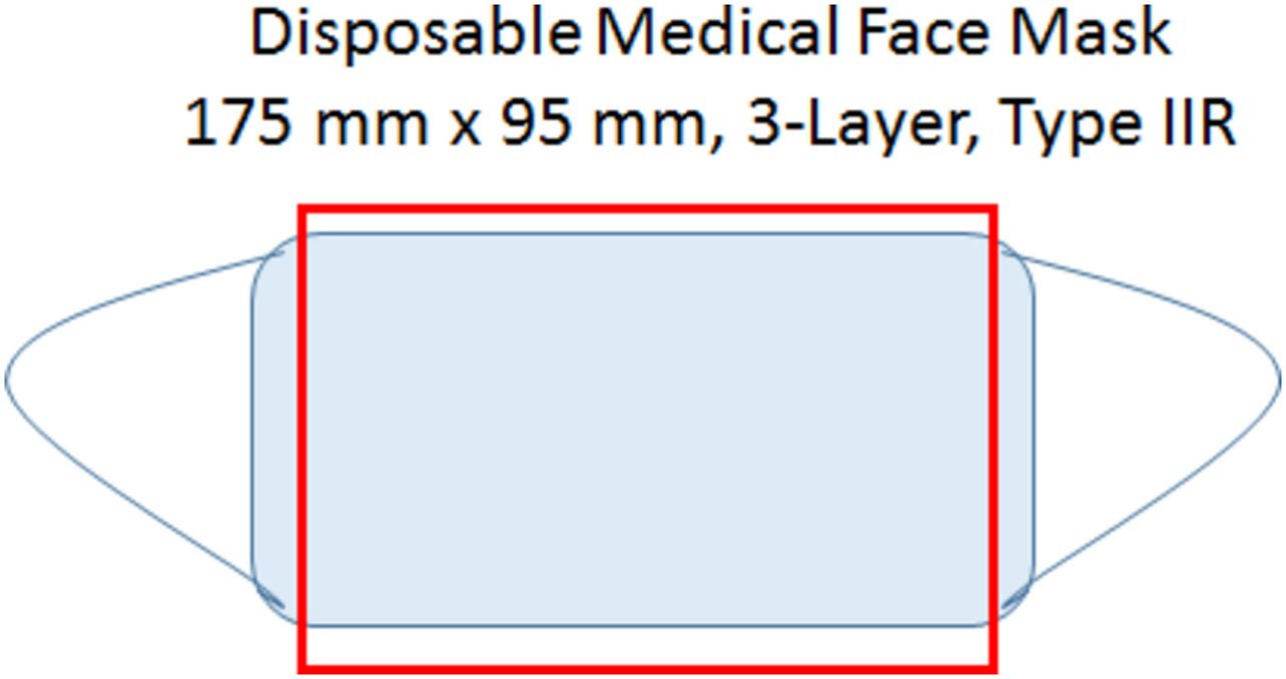
Rose Bengal staining of worn face masks. The area analysed is marked by the red frame. The mask dimensions indicated by the manufactures (175 × 95 mm) exclude folds, which enlarge the surface area.

Staining with Rose Bengal sodium salt was used to detect contamination of masks, as described previously (45). Figure 1 illustrates the area of the mask analysed.

### Microbiological mask study design

In this pilot sample study surgical face masks were collected in March 2022 (during the pandemic obligation) from 15 random willing volunteers (employees of the Gdansk University Department aged 19–65 years), who had worn them for periods from 15 min to 12 h. Wearer details were not further recorded as this did not appear to be crucial for our pilot study, which was intended to show the possible contamination of masks used by the working general population. However, with our random sample, we have captured a realistic usage profile with typical temporal fluctuations due to different users from the general population. Each mask was stored in a separate plastic bag until examination. The masks, excluding the ear loops, were then aseptically cut in several pieces using sterile scissors in a laminar flow cabinet. These pieces were transferred to tubes containing 15 mL of sterile phosphate-buffered saline (PBS), equilibrated for 1 min at room temperature, then vortexed for 30 s. Three unused, clean, surgical masks (Shandong KaiBo Medicinal Packaging Co., Ltd., China) were processed identically as negative controls.

To determine bacterial counts, the suspensions were diluted 10- and 100-fold, then 100-μl volumes were spread on Columbia Agar containing 5% sheep blood (Graso Biotech, Owidz, Poland). Plates were incubated aerobically overnight at 37°C, then colonies were counted. The bacterial load was determined as colony forming units per ml (CFU/mL) of suspension, then rebased as CFU/mask (38). Ten colonies per worn mask were re-plated, grown on Tryptic Soy Broth (Graso Biotech, Owidz, Poland), then stored in 15% glycerol stock solutions (v/v) at −70°C pending molecular identification.

### Identification of isolates by sanger sequencing of the 16S rRNA gene

Forty isolates were identified by PCR and Sanger sequencing of the 16S rRNA gene. Briefly, bacterial colonies were suspended in 30 μL of sterile water and lysed in 95°C, followed by centrifugation at 13,000×*g* for 2 min. The supernates were used for PCR. Primers were: forward F27 5′-AGAGTTTGATCMTGGCTCAG-3′ and reverse R1492 5′-CTACGGYTACCTTGTTACGACTT-3′ (48, 49). The reaction mixture (25 μL) contained: 0.1 μM of each primer, 1 μL of bacterial supernatant, 0.6 U of Taq polymerase (EURx, Gdansk, Poland), 0.2 mM dNTPs and Taq Polymerase buffer (EURx), containing 15 mM MgCl_2_. Cycling conditions involved 94°C for 5 min; 30 cycles of 94°C for 1 min, 50°C for 1 min, 72°C for 1.5 min and a final step at 72°C for 5 min. Sanger sequencing was performed at Macrogen Europe (Amsterdam, The Netherlands) on a 3730xl DNA Analyzer (Thermofisher Scientific, Waltham, MA, USA). PCR amplification was as described by Monciardini et al. (50). The sequencing data were analysed by FinchTV 1.4 (Geospiza, Inc.; Seattle, WA, USA),^1^ the ends of sequenced reads were trimmed, and the resulting assemblies were blasted in the NCBI database. Sequencing data are available in Figshare at https://doi.org/10.6084/m9.figshare.23614797 (accessed on 2 July 2023).

### Biochemical characterisation of isolates

All sequenced isolates were re-plated on Columbia Blood Agar with 5% sheep blood for evaluation of haemolysis, and on Mannitol Salt Agar (Graso Biotech, Owidz, Poland) for the preliminary identification of *Staphylococcus* spp. Staphylococci were further tested using the STAPH LATEX KIT (Prolex™, Pro-Lab Diagnostics, Bromborough, UK) to distinguish *S. aureus* from other species.

### Systematic literature search

We systematically searched for peer-reviewed, scientific studies, up until June 2023, that quantitatively analysed colonisation or contamination of cloth, surgical, N95 and similar masks by bacteria and fungi. The search was performed using PubMed and MEDLINE and included both qualitative and quantitative evaluations. Search terms were created according to the criteria defined in the PICO scheme (51). The non-specific search term “mask” was omitted, as it also includes respirators and anaesthesiologic ventilation masks. Instead, specific terms were chosen: “((face mask) OR (facemask) OR (surgical mask) OR (FFP1) OR (FFP2) OR (FFP3) OR (N95) OR (KF94) OR (KN95)) AND ((microbial contamination) OR (bacteria) OR (fungi)).” Two independent researchers identified and screened eligible studies. Qualitative inclusion criteria were: valid reproducible presentation of the microbial contamination, comprehensible collection of evaluated masks, credibility of the results and clear focus. Quantitative inclusion criteria were: appropriate and precise methods, valid measurement of outcomes, representative selection of evaluated masks and reproducible detection/analytical methods. Selected papers were checked by at least three of the present authors for potential eligibility. Study design, methodology, analytical and experimental methods and outcomes were evaluated. Exclusions and reasons were documented. For included studies, the following data were extracted into tables: author and year, method and type of study, sample size and mask type(s), mask wearing duration(s), outcomes/examined microorganisms, content and main species. Simple mathematical calculations and graphics were performed with Libre-Office Calc, a free and open-source office package from The Document Foundation (52).

## Results

### Abundance and types of bacteria on worn masks

**Figure 2:**
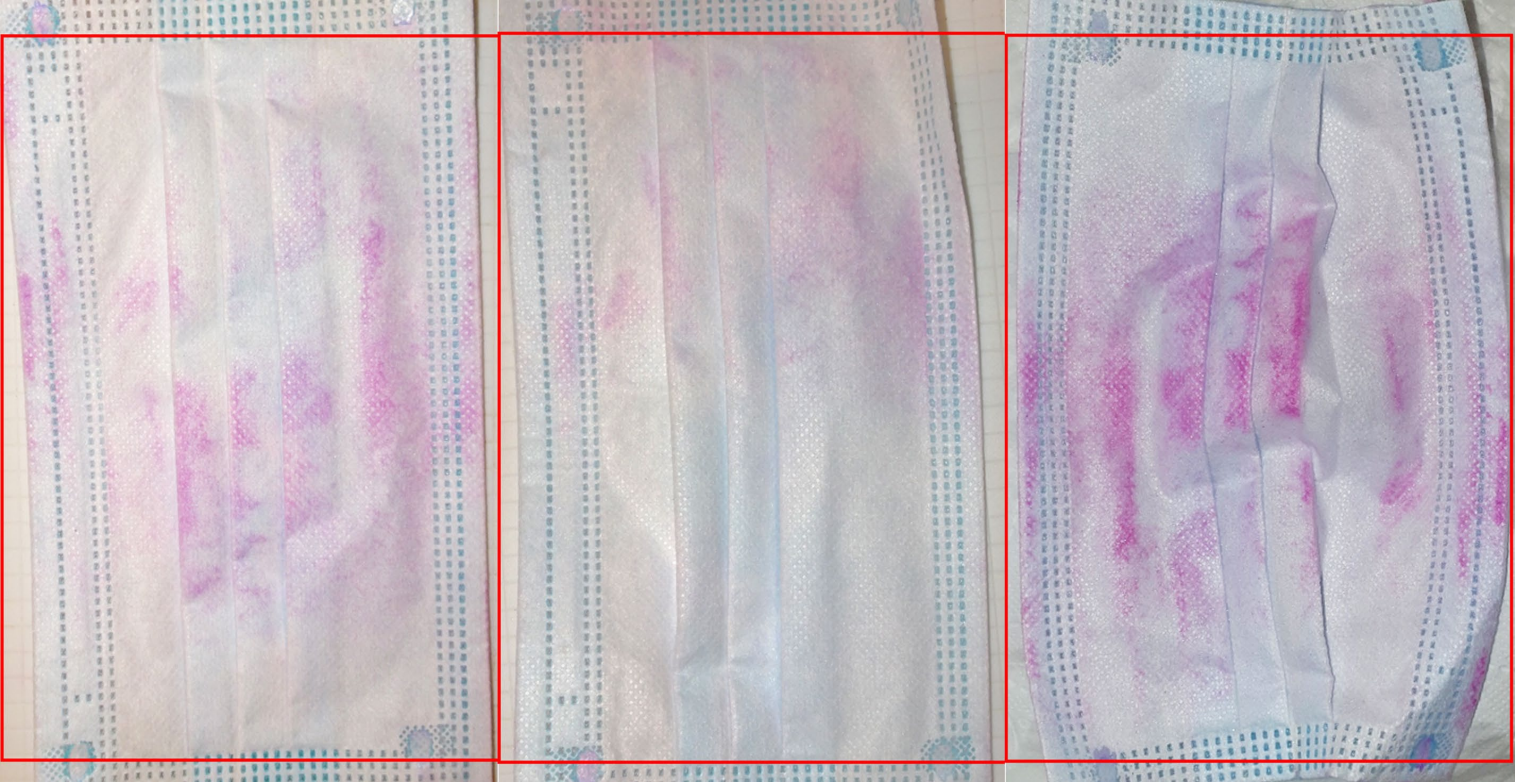
Example masks staining with Bengal Rose, binding to tissue cells, debris and bacteria.

Contamination of worn masks was visible, macroscopically, after staining with Rose Bengal (Figure 2). This dye binds to bacteria, fungi and tissue cells along with debris with the colour intensity suggested to reflect the degree of contamination (53–57).

Based upon culture, the average bacterial load of clean, never-used surgical face mask was 0.1 × 10^3^ CFU recovered/mask whereas the arithmetic mean load on used masks was 4.24 × 10^4^ CFU recovered/mask (geometric mean 1.3 × 10^4^). Bacteria were most abundant on worn masks 5 and 6, with 1.03 × 10^5^ and 2.85 × 10^5^ CFU recovered/mask, respectively (Table 1). Biochemical and molecular identification revealed staphylococcal species on both these latter masks, including *S. aureus*, *S. warneri* and *S. epidermidis* (Supplementary Table 2). Although colony morphology differed between masks, the dominant phenotypes, in almost all cases including the unused masks, were the small white colonies typical of *S. epidermidis* and other coagulase-negative staphylococci (Supplementary Figure 1).

**Table 1:** The abundance of bacteria in masks.

Mask number	Average CFU* recovered/mask and standard deviation**
3 Clean masks	0.1 ± 0.09 × 10^3^
1	4.8 ± 0.3 × 10^3^
2	9.29 ± 0.17 × 10^4^
3	1.35 ± 0.15 × 10^3^
4	9.3 ± 0.3 × 10^3^
5	1.03 ± 0.03 × 10^5^
6	2.85 ± 0.05 × 10^5^
7	1.79 ± 0.14 × 10^4^
8	9.15 ± 0.15 × 10^3^
9	0.45 ± 0 × 10^3^
10	5.55 ± 0.45 × 10^3^
11	3.47 ± 0.29 × 10^4^
12	1.76 ± 0.05 × 10^4^
13	3.45 ± 1.05 × 10^3^
14	1.55 ± 0.05 × 10^4^
15	3.53 ± 0.08 × 10^4^

*CFU, colony forming units; **Standard deviation of three replicates.

### Identification of isolates by sanger sequencing of 16S rRNA gene

Out of 52 colonies subjected to PCR we chose the 40 with the most efficient product amplification for sequencing. Detailed BLAST results are presented in Supplementary Table 1.

The great majority (32, 80%) of these 40 belonged to the genus *Staphylococcus* confirming phenotypic identifications. We identified four coagulase-negative species: *S. epidermidis* (the most abundant), *S. warneri*, *S. pasteuri* and *S. hominis*, all of which belong to the normal human skin and nasal microbiota (Supplementary Table 2) (58). On mask 5 we confirmed coagulase-positive *Staphylococcus* (Supplementary Table 2) along with *S. aureus* and *S. argenteus*.

**Figure 3:**
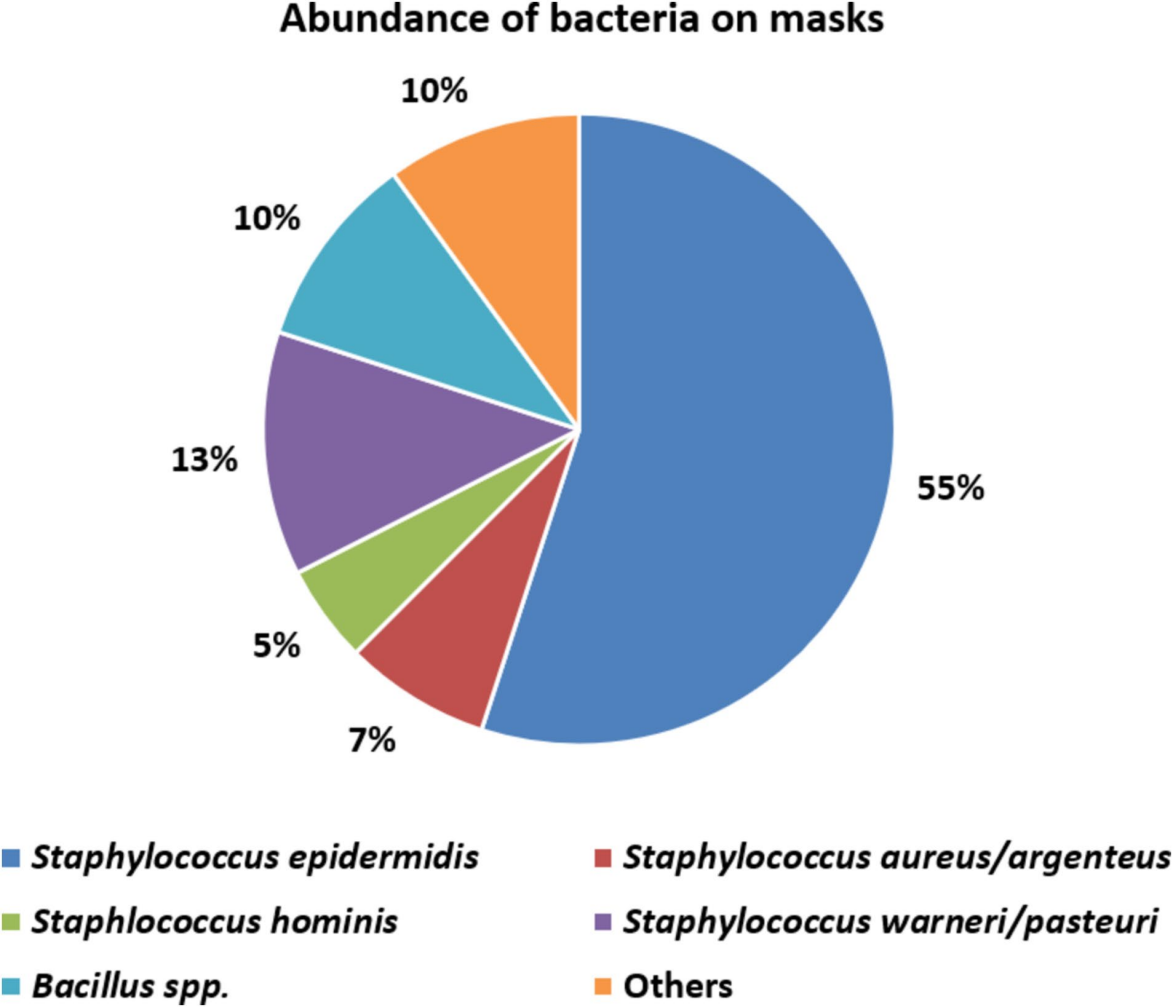
The relative abundance of different bacterial species recovered from masks.

A further four sequenced colonies comprised endospore-forming *Bacillus* species, namely *B. cereus, B. thuringiensis, B. altitudinis, B. megaterium* and others (Supplementary Tables 1, 2), which are soil bacteria (59). Among the four remaining identified colonies (‘Others’ in Figure 3) we found *Sporosarcina newyorkensis*, another endospore-forming Gram-positive rod, occasionally recovered from human bacteraemias and cow’s milk (60). The sole Gram-negative species found was the pseudomonad *Psychrobacter faecalis* (Supplementary Table 2), a psychrophilic species associated with pigeon faeces (61) and reported also from human samples (62). We did not isolate streptococci, although these are a major component of the human oral microbiota. Perhaps, their survival rates on the masks is low, or their recovery requires CO_2_-enriched incubation, not air incubation as used here.

### Biochemical identification of isolates

The same 40 colonies were subjected to biochemical identification, yielding results consistent with the sequencing. Haemolysis was detected for almost all these bacteria (Supplementary Table 2) though its intensity was very variable (Supplementary Table 2; Supplementary Figure 1). Most of the bacteria showed halotolerance but only five fermented mannitol: these latter were tested for coagulase and protein A and three, all from mask 5, proved positive for both characters, confirming identification as *S. aureus* (Supplementary Table 2); all had morphology typical of the species (Supplementary Figure 1, Mask 5).

### Systematic literature search

**Figure 4:**
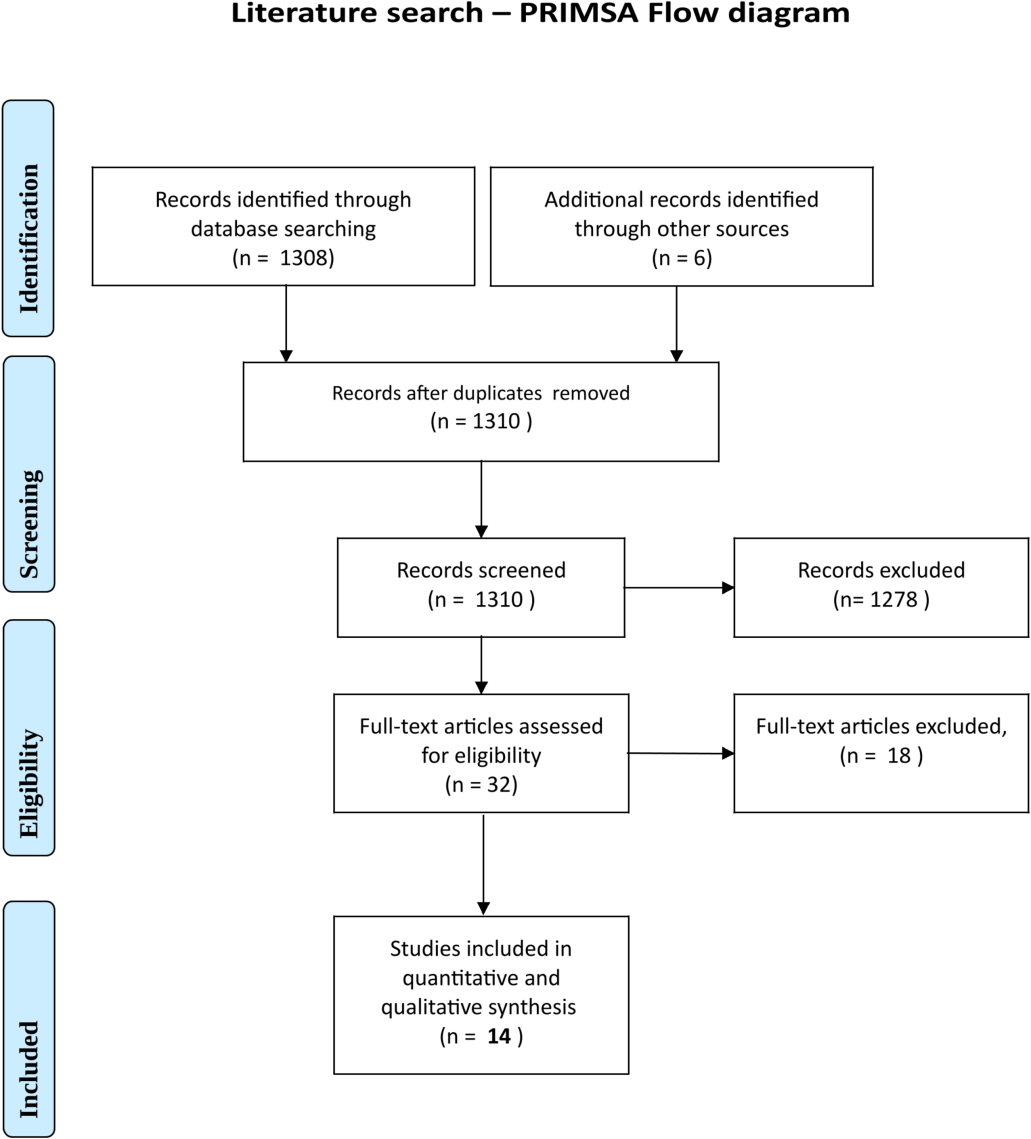
PRISMA flow chart for the literature search.

The literature search initially yielded 1,310 results. This was narrowed (see PRISMA diagram, Figure 4) to 14 studies evaluating bacterial and fungal contamination of cloth, surgical and N95 masks, worn for periods ranging from 5 min to 3 days. Eleven studies considered bacteria, five fungi, and three both (Table 2). Four studies were for the general population whereas 10 were for healthcare workers (HCWs) (38, 41, 42, 44, 46, 63–71). Six were for surgical units (one specifically performing orthopaedic surgery) and five for dental practices (44, 64–67). Only two provided exact quantification and bacterial identification by 16S rRNA; these both investigated the general population (38, 63). Results of the literature search are summarised in the extraction (Table 2).

**Table 2:** Microbiological findings of the literature search (mask contamination with bacteria and fungi).

Author and year	Country	Mask types (population)	Duration of wear	n	Contamination	Method	Maximum level of contamination^*^	Principal microorganisms detected
Present study 2023	Poland	surgical, disposable (general population)	5 min–12 h	15	bacterial (nearly whole mask)	PBS, agar plates 16S rRNA	2.85 ×10^5^ / mask	*Staphylococcus aureus,* *Staphylococcus warneri,* *Staphylococcus epidermidis,* *B. cereus,* *B. thuringiensis,* *B. altitudinis,* *B. megaterium*
Checchi et al. 2022 (67)	Italy	N95 (HCW, dental practice)	30 h	6	bacterial (outer surface samples)	agar plates, eye sighting and counting	10^1^-14^1^ / outer mask area sample	not specified
Delanghe et al. 2021 (38)	Belgium	cotton, surgical (general population)	4 h	21	bacterial (half mask)	PBS, agar plates 16S rRNA	1.53×10^5^ ± 1.96×10^5^ / cotton mask 1.79×10^4^ ± 1.63×10^4^ / surgical mask	*Staphylococcus* spp., *Bacillus* spp., *Acinetobacter* spp.
Gund et al. 2021 (65)	Germany	surgical (HCW, dental practice)	45–60 min	32	bacterial, (external surface samples)	agar plates, MALDI-TOF MS, colony counter	<10^2^ / contact sample external surface	*S. epidermidis,* *S. capitis,* *S. saprophyticus,* *B. cereus*
Gund et al. 2022 (66)	Germany	surgical over N95 (HCW, dental practice)	60–90 min	102	bacterial, (external surface samples)	agar plates, MALDI-TOF MS, colony counter	80 ± 130 / imprint external surface	*Streptococcus,* *Staphylococcus* spp., *Micrococcus* spp., *Bacillus* spp.
Keri et al. 2021 (70)	India	cloth, surgical, N95 (general population)	4–72 h	50	fungi, (inner and outer surface samples)	agar plates, microscopy, lactophenol cotton blue	fungi: 64% outside (32 in 50 masks) 67% inside (14 in 21 masks)	*Aspergillus niger,* *Rhizopus arrhizus,* *Syncephalastrum* spp., *Mucor* spp.
Liu et al. 2018 (46)	China	Surgical (HCW, orthopaedic surgery)	2–6 h	40	bacterial, (first outer layer, second outer layer samples)	agar plates, eye sighting and counting	bacteria: 5.3 (0-2h) / layer samples 7.4 (2-4h) /layer samples 12.8 (4-6h) / layer samples	not specified
Lukasmijarkul et al. 2014 (42)	Thailand	surgical (HCW)	Not given (working day)	203	bacterial, fungal, (outer side and inner side)	agar plates, Gram’s stain, lactophenol cotton blue. microscopic morphology	bacteria: 47 ± 56 / inside area sample 166 ± 199 / outside area sample fungi: 15 ± 9 / inside area sample 34 ± 18 / outside area sample	*Staphylococcus* spp., *Pseudomonas* spp. *Aspergillus* spp., *Penicillium* spp.
Merad et al. 2023 (71)	Algeria	surgical, N95 (HCW)	1–7 h	52	fungal, inner side samples	agar plates, macroscopic and microscopic features of growing colonies	fungi: 88% surgical 8% KN95	*Alternaria* spp. (32%), *Penicillium* spp. (20%), *Aspergillus* spp. (16%)
Monalisa et al. 2017 (44)	India	surgical (HCW, dental practice)	Not given (working day)	36	bacterial, fungal, (outer side and face-side samples)	agar plates, colony counter, biochemical tests	bacteria: 31.7×10^2^ / outer sample 22.8 ×10^5^ / internal sample	*E. coli* (54%), S*. aureus* (25%), *Klebsiella* spp. (5%), *Enterococcus* spp. (4%), *Pseudomonas* spp.(3%), *Enterobacter* spp. (2%), *Candida* (6%), *Aspergillus* spp., *Cladosporium* spp., *Alternaria* spp.
Nightingale et al. 2023 (69)	USA	surgical (HCW)	4–8 h	69	bacterial (whole mask)	selective plates, catalase and coagulase tests	bacterial: 44.9%	*Enterococcus* spp. (44.9%) *S. aureus* (15.9%), *Klebsiella pneumoniae* (14.5%)
Park et al. 2022 (63)	Japan	non-woven, gauze, polyurethane (general population)	1 day (3–6 h) up to 3 days	109	bacterial, fungal, (outer side and face-side samples)	agar plates, 16S rRNA sampling, Gram staining	bacteria: 168.6 ± 24.7 / face-side 36.0 ± 7.0 / outer side fungi: 4.6 ± 1.9 / face-side 6.1 ± 1.9 / outer side	*S. epidermidis,* *S. aureus,* *B. cereus,* *S. saprophyticus* *Aspergillus,* *Microsporum,* *Cladosporium*
Sachdev et al. 2020 (64)	India	surgical (HCW, dental practice)	30 min	240	bacterial, fungal, (outside and inside samples)	agar plates, Gram´s stain, lactophenol cotton blue, microscopic morphology	bacteria: 48±26 / inside mask 180±110 / outside mask fungi: 14±6 / inside mask 32±13 / outside mask	*Staphylococcus* spp. (26.35%), *Pseudomonas* spp. (17.82%), *Streptococcus* spp. (15.50%), *Aspergillus* (6.97%)
Yang et al. 2018 (41)	China	N95 (general population)	5 + 15 min	2	bacterial, (inner surface samples)	agar plates, eye sighting and counting	bacteria: 4.33 (5 min sample) 49 (15 min sample)	not specified
Yousefimashouf et al. 2023 (68)	Iran	surgical, N95 (HCW)	≤2–8 h	175	bacterial, (inner and outer surface samples)	immersion physiological serum, agar plates, analytical profile index kit	Bacteria distribution on 471 positive isolates: 52.2 % (N95) 47.8 % (surgical) counts N95, inner vs outer: 128:118 counts surgical, inner vs. outer: 106:119	Coagulase-negative *Staphylococcus* (28%) *Acinetobacter baumannii* (20.8%), *Pseudomonas aeruginosa* (13.8%), *E. coli* (10.8%), *S. aureus* (10.1%), *ß-Haemolytic Streptococcus* (5.9%), *Enterobacter* (5.4%), *Klebsiella* (3.8%), *Enterococcus* (1.3%)

Grey background = studies in healthcare settings; white background = studies on general population, n—the number of masks tested.

* CFU (Colony Forming Units) per mask, resp. CFU/area, or alternatively percentage of positive tested masks (any contamination found, regardless of level).

HCW, Health care workers; MALDI-TOF MS, matrix-assisted laser desorption/ionisation time of flight mass spectrometry; PBS, phosphate-buffered saline.

## Discussion

We found heavy bacterial contamination of surgical masks worn by the general population, with up to 2.85 × 10^5^ CFU/mask (average 4.24 × 10^4^).

Unfortunately, there are no microbiological standards for worn masks against which to review these findings; in the EU the only relevant bioburden requirement is EN 14683 for new masks, requiring ≤ 30 CFU/g. Nevertheless, since masks amount to a filtering system upstream of the respiratory tract, the limit values for ventilation systems are pertinent, notably the German standard for surfaces of ventilation and air-conditioning, VDI 6022, part 4 (72). This specifies counts of 25 to 100 CFU/25 cm^2^ as ‘borderline’, whilst surfaces with counts > 100 CFU/25 cm^2^ require immediate action or replacement.

A disposable surgical mask has a one-side surface area of ca. 230 cm^2^ (73), meaning that in our worst case (2.85 × 10^5^ CFU/mask = 3.09 × 10^4^ CFU/25 cm^2^), the upper limit of VDI 6022 was exceeded by ca. 310-fold (average 46-fold) (Table 1). Values from a comparable study show 166-fold exceedance with cotton masks (38); another study, for healthcare workers with surgical masks worn for an unspecified period, indicated > 2,000-fold exceedance (Table 2) (44). It should be added that the bacterial burden of a mask lies directly in front of the respiratory tract whereas the vent of an air-conditioning system typically lies several metres away.

The EN 14683 requirements for new masks also were widely exceeded for worn items (Table 2), based upon weights of ca. 3 g for a surgical mask and 4 g for N95/FFP2 masks (74); exceedance of this requirement was evident even for the unworn masks (Table 1).

The heavy general contamination of worn masks was further demonstrable by Rose Bengal staining (Figure 2).

### Bacteria detected: potential clinical implications

Microbiological investigation of used mask predominantly revealed coagulase-negative skin staphylococci and endospore-forming soil bacteria (*Bacillus* spp.) on used (Figure 3). This predominance of staphylococci is in line with other studies on contaminated face masks in the general populace and healthcare workers (42, 44, 64–66, 68). One mask (no. 5) was contaminated with *S. aureus*, a well-known and versatile pathogen (Figure 3; Table 1) (75–78). Up to 30% of the population carry nasal *S. aureus* without symptoms (79) though with an increased risk for autoinfection (75). Contingent contamination of masks may facilitate dissemination of *S. aureus* and, plausibly skin infection (75). An association between nasal carriage and surgical as well as KN95 mask contamination was shown previously for *S. aureus* and even for the non-carriers, the organism was frequently detected on KN95 masks (*p* = 0.04, Fisher’s exact test) implying exogenous sources of contamination (hands, environment and external droplet containing air streams etc.) (75). In support of this, some authors note *S. aureus* contaminates on the external as well as internal surfaces of masks (75).

Several authors have associated the use of face masks skin eruptions, some involving *S. aureus* (80) including new occurrence or exacerbation of acne, rosacea, and seborrhoeic dermatitis (81). Other authors note enrichment of the normal eye microbiota with *S. aureus* from exhaled breath and droplets while wearing a mask contributing to the development of eyelid inflammation (chalazion) (82, 83) and infections of the cornea (84), also deeper eye infections in the context of treatments (endophthalmitis following vitrectomy) (85). There is also some evidence that *S. aureus* can increase replication of the SARS-CoV-2 virus by 10- to 15-fold (86), though this seems more pertinent in the upper nose than on a mask, where the virus is unlikely to be replicating.

Among other staphylococci, we predominantly found *S. epidermidis* (Figure 3). On one hand this is a normal and harmless component of the skin microbiota; on the other, it may be a hazard for vulnerable immunosuppressed individuals (87–89). Even in healthy individuals, coagulase-negative staphylococci, at high abundance, may contribute to inflammatory skin conditions such as atopic dermatitis and acne vulgaris (58, 90–92) with evidence that wearing a mask significantly increased the incidence of acne in particular (93–101).

We also found *Bacillus* spp. in the masks, including species that produce enterotoxins (59). Although bacterial growth in masks may be possible (see below) we saw no evidence that growth attained the levels—typically >10^6^/g—associated with toxins in food (102). Moreover, wearers (except maybe children) are unlikely to chew on their masks, meaning that these organisms can be dismissed as a risk.

### Literature review on mask contamination

**Figure 5:**
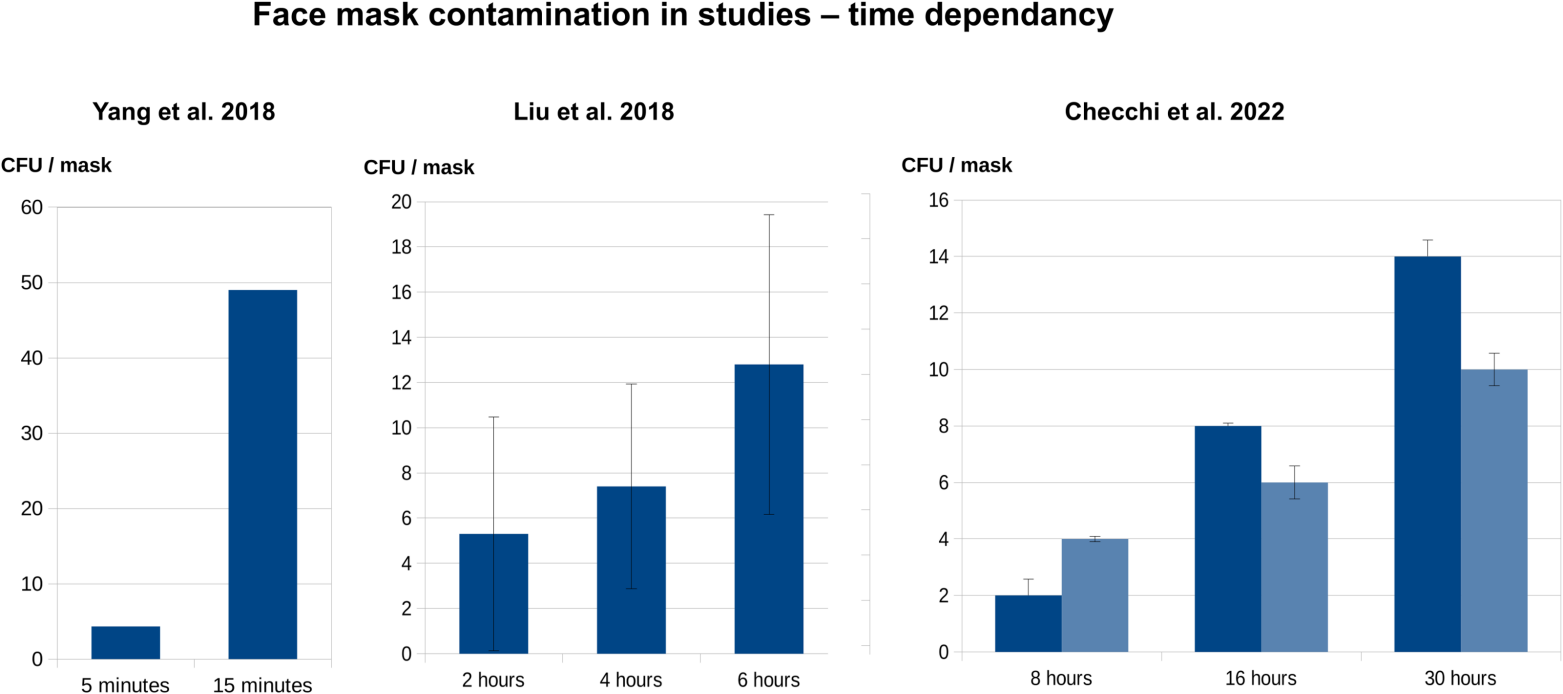
Time dependency of face mask contamination during wear, based upon literature data (Table 2). The diagrams indicate the association between CFU/mask and wearing duration, based on mean values from three publications (41, 46, 67). If included in the primary studies, the standard deviations are also shown. Yang et al. investigated the inner surfaces of masks worn by the general population, whereas both Liu et al. and Checchi et al. examined the outer layers of masks worn by HCW.

Our literature review showed that all relevant mask types (surgical, N95, cloth) become increasingly contaminated with microorganisms during wear (Table 2; Figure 5) (38, 40, 41, 46, 65, 67, 71).

The literature reports contamination by bacteria of the genera Acinetobacter, Bacillus, Escherichia (specifically, *E. coli*, a faecal organism), Enterobacter, Enterococcus (another faecal organism), Klebsiella (including *K. pneumoniae*), Micrococcus, Pseudomonas, Staphylococcus (including *S. aureus*) and Streptococcus and by fungi of the genera Aspergillus, Alternaria, Candida, Cadosporium, Microsporum and Mucor (Table 2). These organisms are nourished by human saliva, nebulised oral biofilm and exhaled breath condensate, creating an underestimated biosafety concern.

In the general population, internal mask contamination typically exceeds external for bacteria—and perhaps, although this varies with the study—also for fungi (Table 2) (63, 70). For the healthcare workers using surgical masks, on the other hand, external contamination exceeds internal contamination both for bacteria and fungi (*p* < 0.001) (42, 44, 64) and correlates with microbiological air quality in the areas where these staff were working (42). For N95 masks, however, internal bacterial contamination appears higher than external even in healthcare settings (68). Moreover, the total bacterial contamination of worn N95 masks exceeded that of similarly worn surgical masks (68).

Fungal contamination is seen up to 70–88% of used masks (70, 71), and can be also higher inside than outside the mask (70). This is surprising, given that fungi must come from outside the mask (63).

A comparison of maximal bacterial face mask counts for healthcare workers and the general population, based on Table 2 data and wearing/using times between 5 min and 3 days, showed a high variance in data due to the variance in wearing times and users and environmental factors. There is a tendency for higher bacterial loads in the general population (Table 2). These findings may reflect wider inappropriate and extended usage in the general population (7, 8). Due to the small number of similar studies, a meta-analytical statistical evaluation was not carried out.

### Face mask contamination—contributing factors

Masks are a good matrix for microbial accumulation and potentially, growth, retaining an above-ambient temperature (103–107), moisture, and nutrient-rich debris (38–41, 45, 108). Besides substances sucked in from the outside, nutrients comprise exhaled proteins and other debris, exfoliated and dead epithelial cells. Condensing droplets in the exhaled breath contain non-volatile metabolites, salts, lipids and proteins along with intact and degraded bacteria and viruses (109). This organic richness was visualised in our Bengal Rose staining. Growth, rather than mere survival (38, 39, 41, 45, 108, 110) of bacterial and fungal colonies is revealed by scanning electron microscopy of face masks (FFP2) worn for several hours (40).

The dead-space of rigid N95 masks provide a particularly warm, wet environment (103) with a relative humidity 1.5–2.6 times higher than externally (41) rising to 100% after 60 min of use (40). This may create a particularly attractive breeding ground for bacteria (41) explaining the findings (above) that the N95 masks become more heavily contaminated than surgical masks and that, in healthcare settings, internal contamination exceeded external, reversing the pattern seen for surgical masks (68).

Microorganisms trapped and incubated in the mask may be distributed to the wearer, the environment and to others (16, 111–113). If leakage, owing to defect or poor fit, affects 1% of the mask area, the filtration efficiency is reduced by 50%; if the gap is 2% of the mask area, efficiency is reduced by 75% (114). Moreover, the exhalation filtration efficiency is significantly lower than the theoretical filtration efficiency—being 12.4 and 46.3% for surgical and N95 masks, respectively (115). In operating theatres, the recommended wearing duration is limited to few hours (116) as surgical masks lose effectiveness over time (117). Whereas a fresh mask almost completely prevented bacterial contamination of an agar plate held 10–12 cm from the mouth, this effectiveness was measurably reduced within 30 min and negligible after 2 h (118). This brief period of filtration efficiency was further reduced if the mask was poorly fitted (114, 119) or wetted (119).

Penetration of microorganisms between mask layers is possible, through capillary action depending on humidity and the specific organisms among other factors (120). This in turn, may facilitate the formation of tiny organism-laden droplets. These then may be projected or inhaled with every breath (16, 111, 114, 115, 121–123). In this context, we underscore the predominantly oral breathing while wearing a mask (16, 124), in contrast to normal unimpeded breathing, which is largely via the nose, with greater filtration. Oral breathing increases the hazard of directly inhaling microorganisms from the mask into the deeper airways (125). In a human study with a radiolabelled aerosol and average particle diameters of 4.4 μm (range 3.8–5.1 μm) scientists found a large increase in deposition in the lungs (+37%) when breathing orally compared with via the nose (75% vs. 38%) (126). Additionally, masks—and especially the N95 type—impair natural mucociliary clearance of the upper airways, further enhancing inhalation and distribution of bacteria (127).

Finally, in context, face masks contain plastics, to which microorganisms can adsorb (40, 128). Consequently, as well as aerosols, plastic micro-particles may also be released by masks (129–133), acting as carriers for the distribution of pathogenic bacteria and fungi (134). Interestingly, there is hardly any surface or material, not even the bare skin, that ensures such survival and long-term preservation of infectivity for the viruses as the plastic-polypropylene network of the masks, in which SARS-CoV-2 viruses are stored and remain infectious for up to 2 weeks—even when dried (135).

### Face mask contamination—potential clinical implications

In a pre-COVID cross-sectional study on 710 individuals, the wearing (for religious reasons) of cloth facial coverings by Saudi women, drawn from the general population, was associated with statistically increased incidences of ‘common cold’ and asthma (17). Elsewhere, pathophysiological skin changes (136) were associated with mask wearing in the general population and healthcare workers (137, 138). Several authors found changes in skin metabolomics, with an increased risk of barrier disruption and inflammation, putatively owing to dysbioses of the skin microbiome (136, 139, 140) leading to—or promoting development of—atopic dermatitis and acne vulgaris (139). In context N95 respirators caused a more significant disorder than surgical masks (139).

Eye conditions also have been associated with mask use (82–85, 121, 141–145), whilst Islam et al. found indirect evidence of changes in the oral microbiome (146). Sukul et al. changes in the gut microbiome (metabolic alterations) (19) whilst Xiang et al. found change of the nasal microbial communities after prolonged mask wearing (110). Lastly, face masks are mentioned as possible factors behind an increase in mucormycosis cases during the COVID-19 pandemic particularly in immunocompromised or otherwise vulnerable individuals (70, 71, 147).

### Practices for minimising microbial contamination

There are general considerations for the use of face masks in any situation, along with official advice on their proper use (16, 129, 148). Minimising microbial contamination is critical to ensure their safe use, especially in healthcare. The WHO recommends to avoid touching the mask surface, also that masks should be stored in a clean, dry place away from potential contaminants (6). Disposable masks should be removed after each use and not reused. Training should be provided on how to put on and take off masks so as to prevent microbial spread and self-infection. The WHO further recommends cleaning hands before touching a mask (both before and after removing it). When the mask is removed, it should be stored in a clean plastic bag or disposed of in a waste garbage can (6).

In some situations, a face shield can be used in conjunction with masks to provide an additional barrier against contamination. Lastly, the mask should be worn for as short a time as possible, not only for microbiological reasons (time-dependent contamination of the face mask during wearing), but also for toxicological and physio-metabolic reasons (14, 129).

It is self-evident that large sections of the population, including children, are unable to follow these complex instructions adequately and consistently (148). Alternatives to masks should be researched and prioritised (e.g., ventilation systems, hygiene measures and other).

### Findings in context

Long before the pandemic, face masks became widely used in medicine (notably surgery) healthcare and some manufacturing industries (16, 149–151), aiming to prevent or minimise infection or contamination (8, 14, 73, 151–159). Nevertheless, their effectiveness in healthcare settings was debatable long before 2020 (160) and their role in the operating theatre remains controversial (161). Given this history, there has been surprisingly little research on the effects of long-term usage by professional groups. Although masks filter larger debris and aerosol droplets from the air, they carry the microbiological risks outlined here along with toxicological, physiological, psychological and sociological harms (14, 16, 18–35, 129, 162).

The risks and benefits of requiring mask use by populations must be weighed from ethical and medical standpoints (13, 14, 16, 163, 164). For masks to be demanded, the side effects and risks must be lower than the risk of not wearing a mask. A gold-standard Cochrane evaluation, based on clinical trials (12) found no substantive evidence of efficacy in preventing viral respiratory infections and one recent study, albeit with several possible confounders, even found mask-wearing to be associated with an increased risk of COVID-19 infection (165). On the other hand, the potential harms are numerous (2, 3, 5, 14–16, 19–23, 36, 37, 166–172). They include MIES (16), harmful blood-gas alterations (14, 19) and the potential microbiological hazards outlined here. Masks should not be mandated for the general population given this balance of evidence against their use. These points have been raised by many scientists (14, 16, 17, 36, 37, 129, 166, 173–175) including leading breathing experts (176).

## Limitations and strengths

The strengths of our paper are the use a precise method—16S rRNA sequencing—to identify the bacteria found. In addition, we undertook a systematic literature overview and discuss the results from holistic microbiological and clinical perspectives. The masks collected in our study were provided by random individuals during daily life, representing a realistic general population sample. Rose Bengal staining strikingly visualised extensive contamination. Both, our limited sample size and rapid literature review should be seen only as a pilot assessment, with further analysis needed. Due to the small numbers of studies of same design, a meta-analysis was not carried out. Rather the strength of this review is qualitative, cataloguing the extensive scientific literature published by many scientists worldwide over several decades, demonstrating experimental evidence of face mask contamination and its risks.

## Conclusion

Both our experimental study and the published literature show that face masks accumulate microorganisms, including pathobionts (Tables 1, 2) (38, 41, 42, 44, 46, 63–71, 177), with a microbial load up to several hundred times higher than the German standard VDI 6022 limit for ventilation systems surfaces (72) and the EN 14683 requirements for unused masks. Contamination increases with extended wearing time (Figure 5) (38, 41, 46, 65–67, 70, 71) and is greater for N95 than surgical masks (68). Most contamination was with staphylococci, occasionally including the pathogen *S. aureus*.

Put simply: (i) the mask act as a filter trap with bacteria accumulating on its external and internal surfaces; (ii) the mask then acts as a “microbiological incubator” at the entrance of the airways; (iii) microorganisms may grow within the mask, nourished by skin debris, mucus and “exhaled breath condensate” (16, 38, 39, 41, 45, 108–110). These trapped organisms/pathogens then may be inhaled, promoting infection of the respiratory tract (17, 37) or, when distributed via air streams (111, 114, 115, 122, 142, 143, 178, 179) the eye (82–85, 121, 142). In addition, the skin microbiome is disrupted, potentially leading to or promoting other infections and allergic conditions (38, 77, 110, 140, 180).

Lastly, accumulated microorganisms may be distributed via leakage (111, 114, 115), amplified by the atomiser effect of the mask (14, 16, 122, 181, 182).

A Cochrane analysis, based solely on the highest grade of evidence, found no evidence that masks reduced the spread of respiratory viral infections in the general population (12). On the other hand, their detriments, over and above those investigated here, are clear. They impede communication (32–34, 94, 183–188). They impede learning, especially for children (2, 3, 5, 14, 26, 35, 148, 162, 171, 174, 177, 189). They are associated with transient hypoxaemia (decreased blood O_2_), transient hypercarbia (increased blood CO_2_) (14, 16, 19, 21–23, 171, 172). They deny the wearer of the most basic individuality—of showing their face (26, 27, 30–34, 162, 189). Their long-term imposition is especially harmful for vulnerable members of the population (14, 16, 19). Recent scientific papers indicate toxicological issues via inhalation of plastic particles and cancerogenic organic compounds originating from the mask material (14, 18, 129, 133).

In short, the adverse effects of masks are clear (2, 3, 5, 16, 18, 19, 23, 36, 129, 166–172, 190), whereas the protective antiviral effect in real life scenarios remains doubtful (12–15, 165, 175, 191–209). Given this, together with the microbiological contamination issues highlighted, masking laws and requirements do not meet the basic medical ethic of ‘Do no harm’. Laws and mandates requiring mask use accordingly have no valid place in respiratory pandemic management.

## Data Availability

The datasets presented in this study can be found in online repositories. The names of the repository/repositories and accession number(s) can be found in the article/Supplementary material.

## Footnotes

^1^ http://www.geospiza.com
