# Supplementary material for: The bacterial burden of worn face masks—observational research and literature review
Source: Front Public Health. 2024 Dec 3;12:1460981. doi: 10.3389/fpubh.2024.1460981 (PMC11649673; doi:10.3389/fpubh.2024.1460981)
Supplement: Supplementary file 1 [file Data_Sheet_1.pdf]

SUPPLEMENT

The bacterial burden of worn face masks—observational research and literature review

Kai Kisielinski , Barbara Wojtasik, Aleksandra Zalewska, David M. Livermore and Agata Jurczak-Kurek

DOI 10.3389/fpubh.2024.1460981

**Supplementary Figure 1**	page 2-3
**Supplementary Table 1**	page 4-5
**Supplementary Table 2**	page 6-8

Supplementary Figure 1.

Bacterial colonies isolated from 15 randomly collected surgical face masks on blood agar.

Mask 1

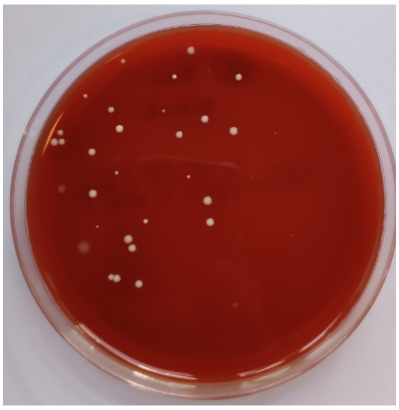

Mask 2

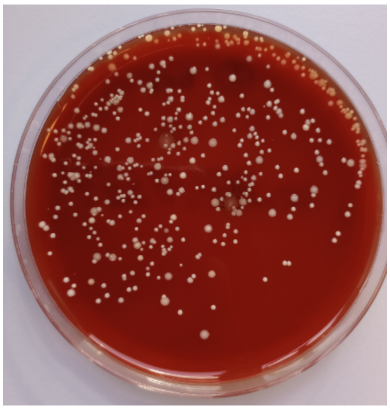

Mask 3

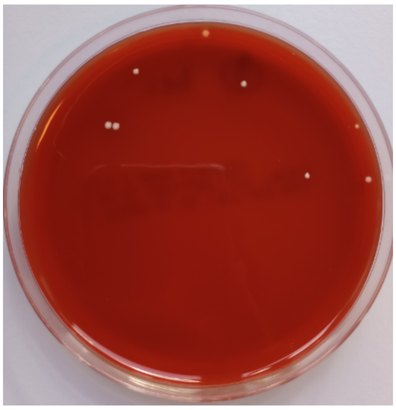

Mask 4

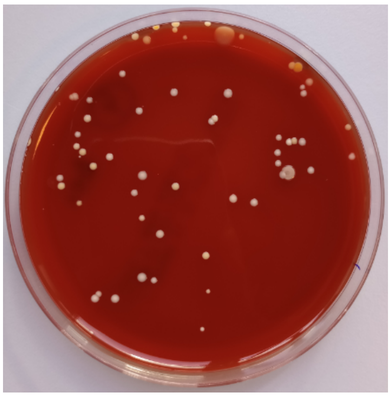

Mask 5

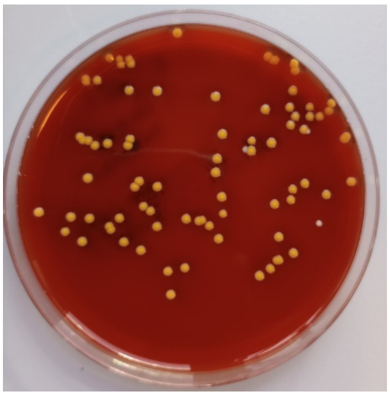

Mask 6

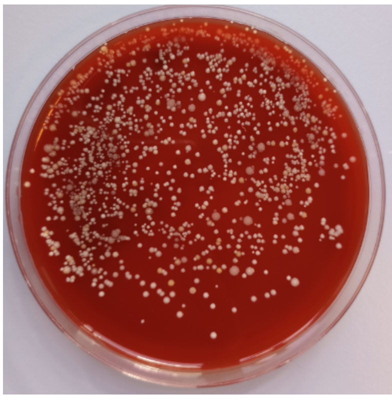

Mask 7

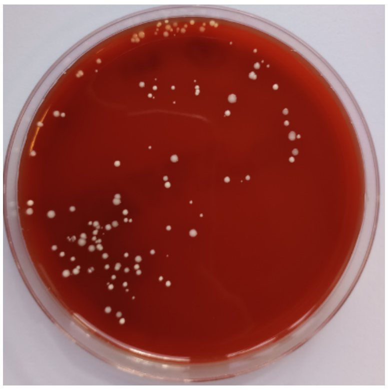

Mask 8

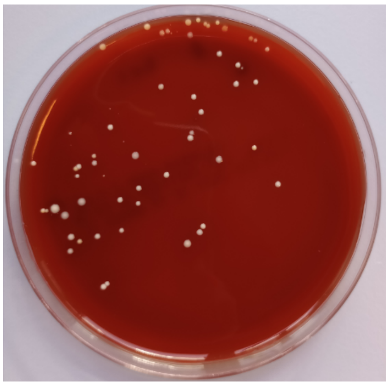

Mask 9

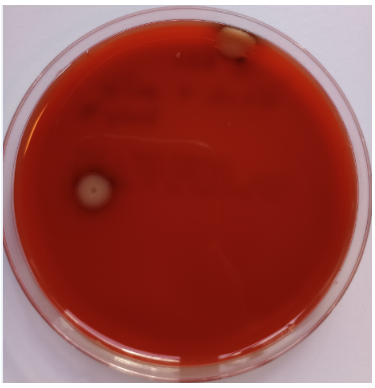

Mask 10

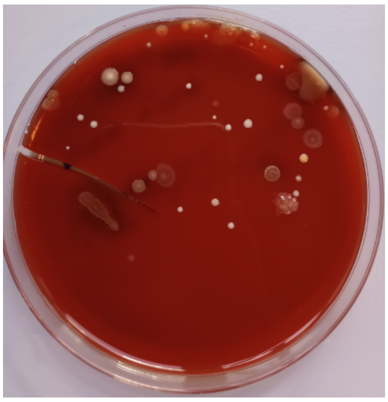

Mask 11

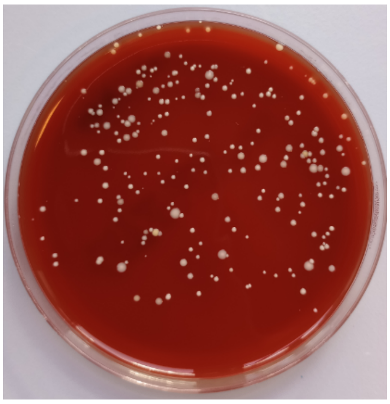

Mask 12

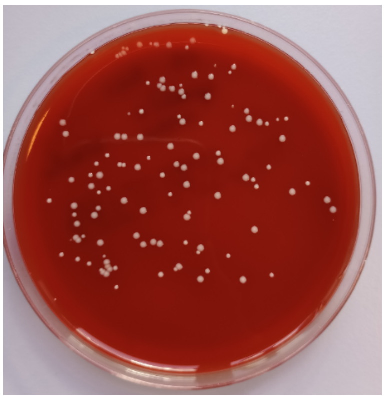

Mask 13

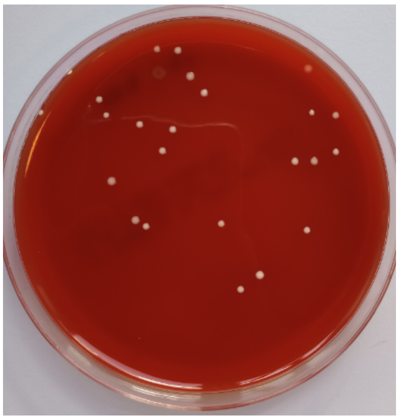

Mask 14

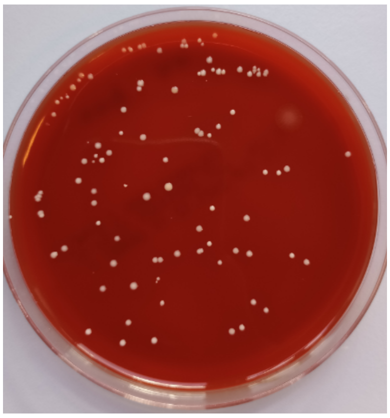

Mask 15

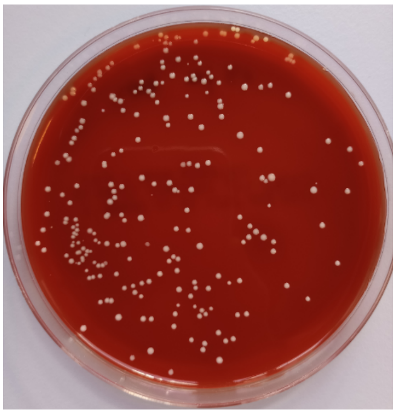

Supplementary Table 1.

BLASTn results of 16S rRNA-based identification of isolated bacteria from masks.

	Mask number	Clone number	Seqeunce code	Trimmed sequence range	Sequence length	The highest identity record	Coverage (%)	Identity (%)	Genome accession number of the first highest identity record
1	1	2	21CDZAD040	C:10-C:1095	1086	*Bhargavaea ginsengi*	100	100	KT719934.1
2	1	5	21CDZAD020	C:15-A:1078	1064	*Staphylococcus epidermidis*	100	100	CP119047.1
3	1	6	21CDZAD014	C:13-A:806	794	*Staphylococcus warneri/pasteuri*	100	100	MK203007.1
4	2	1	21CDZAD049	T:10-A:1074	1065	*Staphylococcus epidermidis*	100	100	CP065656.1
5	2	2	21CDZAD039	C:16-A:976	961	*Staphylococcus epidermidis/warneri*	100	100	CP040883.1
6	2	4	21CDZAD005	C:9-T:1050	1042	*Staphylococcus epidermidis*	100	100	CP040883.1
7	3	1	21CDZAD029	C:16-A:1142	1127	*Staphylococcus epidermidis*	100	100	CP040883.1
8	3	6	21CDZAD013	C:15-T:806	792	*Staphylococcus epidermidis*	100	100	MT482624.1
9	4	2	21CDZAD038	A:31-T:1121	1091	*Staphylococcus epidermidis*	100	100	MT613456.1
10	4	3	21CDZAD035	A:35-T:675	641	*Staphylococcus warneri/pasteuri*	100	100	MT642942.1
11	4	5	21CDZAD012	C:17-T:882	866	*Staphylococcus epidermidis*	100	100	MT482624.1
12	5	1	21CDZAD048	G:33-C:995	963	*Staphylococcus aureus/argenteus*	100	100	CP119113.1
13	5	3	21CDZAD026	A:36-A:1123	1088	*Staphylococcus aureus/argenteus*	100	100	CP119113.1
14	5	4	21CDZAD025	T:17-A:1121	1105	*Staphylococcus aureus/argenteus*	100	100	CP119113.1
15	6	1	21CDZAD037	C:14-C:1151	1138	*Staphylococcus warneri/pasteuri*	100	100	OP263325.1
16	6	2	21CDZAD032	C:14-A:1120	1107	*Staphylococcus epidermidis*	100	100	MT604781.1
17	7	1	21CDZAD028	C:19-A:1082	1064	*Staphylococcus epidermidis*	100	100	CP119047.1
18	7	3	21CDZAD027	C:16-A:1079	1064	*Staphylococcus epidermidis*	100	100	CP119047.1
19	8	1	21CDZAD047	C:16-C:1043	1028	*Staphylococcus hominis*	100	100	MT544813.1
20	8	2	21CDZAD031	C:15-A:1147	1133	*Staphylococcus epidermidis*	100	100	CP024408.1
21	9	1	21CDZAD046	C:10-G:981	972	*Paenibacillus lemnae*	100	99.07	MN581184.1
22	9	2	21CDZAD030	C:19-C:1039	1021	*Bacillus* spp.*	100	100	OQ509998.1
23	9	4	21CDZAD011	G:25-C:1087	1063	*Bacillus* spp.*	100	100	OQ534872.1
24	10	1	21CDZAD045	A:25-T:1030	1006	*Staphylococcus epidermidis*	100	100	MT605363.1
25	10	2	21CDZAD024	C:14-A:1077	1064	*Staphylococcus epidermidis*	100	100	CP119047.1
26	10	7	21CDZAD016	A:22-A:1025	1004	*Psychrobacter faecalis/pulmonis*	100	100	MT269580.1

27	10	10	21CDZAD015	T:19-G:1031	1013	*Staphylococcus epidermidis*	100	100	KY623304.1
28	11	1	21CDZAD044	T:14-A:1063	1050	*Staphylococcus epidermidis*	100	100	MT604781.1
29	11	4	21CDZAD019	T:13-G:916	904	*Staphylococcus epidermidis*	100	100	MT445298.1
30	11	6	21CDZAD010	T:10-T:795	786	*Staphylococcus warneri/pasteuri*	100	100	MT642942.1
31	12	2	21CDZAD036	C:16-A:995	980	*Staphylococcus epidermidis*	100	100	CP040883.1
32	12	5	21CDZAD009	C:12-C:1139	1128	*Staphylococcus epidermidis*	100	100	MT482624.1
33	12	7	21CDZAD008	T:24-A:1089	1066	*Staphylococcus epidermidis*	100	99.91	KY623304.1
34	13	1	21CDZAD043	T:15-A:973	959	*Staphylococcus hominis*	100	100	CP054883.1
35	13	2	21CDZAD034	C:13-C:1125	1113	*Staphylococcus warneri/pasteuri*	100	100	MT642942.1
36	14	1	21CDZAD042	G:24-T:1120	1097	*Bacillus altitudinis*	100	99.91	OM670223.1
37	14	2	21CDZAD018	T:21-G:1033	1013	*Bacillus spp.***	100	100	MT605432.1
38	14	3	21CDZAD017	T:20-C:993	974	*Sporosarcina newyorkensis*	100	100	LN774518.1
39	15	1	21CDZAD041	C:16-T:1123	1108	*Staphylococcus epidermidis*	100	100	MK425675.1
40	15	4	21CDZAD021	C:22-A:1126	1105	*Staphylococcus epidermidis*	100	100	CP119047.1

**Bacillus* spp: *B. thuringiensis, B. tropicus, B. cereus, B. proteolyticus, B. albus, B. toyonensis, B. subtilis, B. paranthracis, B. mobilis, B. wiedmanni,*

**Bacillus spp.: *B. megaterium, B. aryabhattai, B. acidiceler, B. zanthoxyli*

Supplementary Table 2.

Molecular identification of bacteria isolated from surgical masks, and their selected biochemical characteristics.

Mask number	Clone number	Haemolysis*	Salt tolerance (6.5 % NaCl)	Mannitol fermentation	Coagulase	Result of molecular identification based on 16S rRNA
1	2	-	+	-	-	*Bhargavaea ginsengi*
	5	+	+	-	-	*Staphylococcus epidermidis*
	6	+	+	+	-	*Staphylococcus warneri/pasteuri*
2	1	++	+	-	-	*Staphylococcus epidermidis*
	2	++	+	-	-	*Staphylococcus epidermidis/warneri*
	4	++	+	-	-	*Staphylococcus epidermidis*
3	1	++	+	-	-	*Staphylococcus epidermidis*
	6	+	+	-	-	*Staphylococcus epidermidis*
4	2	+	+	-	-	*Staphylococcus epidermidis*
	3	+	+	+	-	*Staphylococcus warneri/pasteuri*
	5	++	+	-	-	*Staphylococcus epidermidis*

5	1	+	+	+	+	*Staphylococcus aureus/argenteus*
	3	+	+	+	+	*Staphylococcus aureus/argenteus*
	4	+	+	+	+	*Staphylococcus aureus/argenteus*
6	1	+	+	-	-	*Staphylococcus warneri/pasteuri*
	2	++	+	-	-	*Staphylococcus epidermidis*
7	1	+	+	-	-	*Staphylococcus epidermidis*
	3	+	+	-	-	*Staphylococcus epidermidis*
8	1	+	+	-	-	*Staphylococcus hominis*
	2	+	+	-	-	*Staphylococcus epidermidis*
9	1	-	-	-	-	*Paenibacillus lemnae*
	2	++	-	-	-	*Bacillus* spp.
	4	++	-	-	-	*Bacillus* spp.
10	1	+	+	-	-	*Staphylococcus epidermidis*
	2	+	+	-	-	*Staphylococcus epidermidis*
	7	-	-	-	-	*Psychrobacter faecalis/pulmonis*
	10	++	-	-	-	*Staphylococcus epidermidis*
11	1	+	+	-	-	*Staphylococcus epidermidis*

	4	+	+	-	-	*Staphylococcus epidermidis*
	6	+	+	+	-	*Staphylococcus warneri/pasteuri*
12	2	+	+	-	-	*Staphylococcus epidermidis*
	5	++	+	-	-	*Staphylococcus epidermidis*
	7	++	+	-	-	*Staphylococcus epidermidis*
13	1	+	+	-	-	*Staphylococcus hominis*
	2	+	+	+	-	*Staphylococcus warneri/pasteuri*
14	1	++	+	+	-	*Bacillus altitudinis*
	2	-	+	+	-	*Bacillus* spp.
	3	-	-	-	-	*Sporosarcina newyorkensis*
15	1	+	+	-	-	*Staphylococcus epidermidis*
	4	+	+	-	-	*Staphylococcus epidermidis*

Legend: ***** ++ intense haemolysis, + haemolysis visible under colonies,

both salt tolerance and mannitol fermentation were measured in Mannitol Salt Agar.
